# Role of Serum Amyloid A in Abdominal Aortic Aneurysm and Related Cardiovascular Diseases

**DOI:** 10.3390/biom11121883

**Published:** 2021-12-15

**Authors:** Preetha Shridas, Avery C. Patrick, Lisa R. Tannock

**Affiliations:** 1 Department of Internal Medicine, University of Kentucky, Lexington, KY 40536, USA; Lisa.Tannock@uky.edu; 2 Saha Cardiovascular Research Center, University of Kentucky, Lexington, KY 40536, USA; 3 Barnstable Brown Diabetes Center, University of Kentucky, Lexington, KY 40536, USA; 4 Pharmacology and Nutritional Sciences, University of Kentucky, Lexington, KY 40536, USA; avery.patrick@uky.edu; 5 Veterans Affairs Lexington, University of Kentucky, Lexington, KY 40536, USA

**Keywords:** cardiovascular disease, abdominal aortic aneurysm, serum amyloid A, HDL

## Abstract

Epidemiological data positively correlate plasma serum amyloid A (SAA) levels with cardiovascular disease severity and mortality. Studies by several investigators have indicated a causal role for SAA in the development of atherosclerosis in animal models. Suppression of SAA attenuates the development of angiotensin II (AngII)-induced abdominal aortic aneurysm (AAA) formation in mice. Thus, SAA is not just a marker for cardiovascular disease (CVD) development, but it is a key player. However, to consider SAA as a therapeutic target for these diseases, the pathway leading to its involvement needs to be understood. This review provides a brief description of the pathobiological significance of this enigmatic molecule. The purpose of this review is to summarize the data relevant to its role in the development of CVD, the pitfalls in SAA research, and unanswered questions in the field.

## 1. Introduction

### The SAA Family

**Figure 1:**
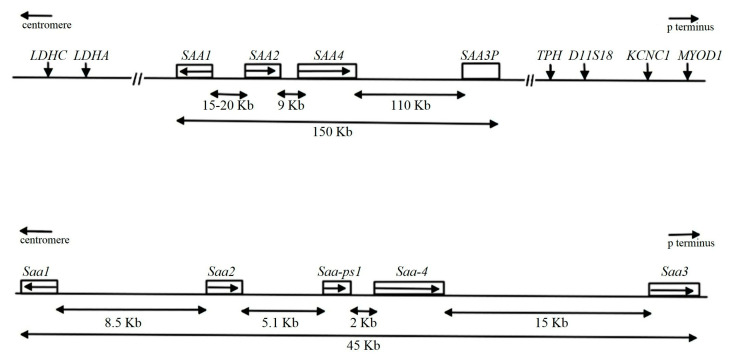
Map of the human and mouse SAA gene families. The human family spans 150 kb on chromosome 11p15.1, and the mouse family spans 45 kb on chromosome7p. The relative positions of flanking genes are indicated in the human cluster. Arrows within SAA genes indicate 5′→3′ orientation of the gene. The human genes (top), SAA1 and SAA2 encode two major acute-phase proteins, and SAA3 (SAA3P) is a pseudogene. SAA4 encodes a constitutively expressed protein. MouseSaa1 (designated Saa1.1), Saa2 (designated Saa2.1), and Saa3 encode three acute-phase SAA isoforms. Mouse Saa4 encodes a constitutively expressed SAA isoform and is present at lower levels (bottom).

Serum amyloid A (SAA) proteins are a family of low molecular weight proteins of 104–112 amino acid residues first described almost 50 years ago [1]. There are four SAA genes in the human genome, of which two are acute-phase proteins, SAA1 and SAA2. One is a pseudogene (SAA3); however, in mice and other species, SAA3 is expressed and is an acute-phase protein [1,2], and one is expressed constitutively (SAA4). The SAA1 and SAA2 genes are coordinately regulated and are arranged ‘head-to-head’ in a gene cluster, which also contains SAA3 and SAA4 on chromosome 11p15.1 and on chromosome 7 in human and mouse, respectively, as shown in Figure 1 [3,4,5]. SAA is remarkably conserved in mammalian evolution. Human SAA1 and SAA2 are 96% homologous over their entire length and correspond to mouse SAA1.1 and SAA2.1. Mice encode and express full-length SAA3 [6]. Murine SAA1.1 and SAA2.1 are 91% identical to each other and approximately 67% identical to murine SAA3. Mouse SAA3 shares 70% amino acid identity with human SAA1 [7]. While SAA3 is thought to exert functional effects similar to the other two human and mouse SAA isoforms [8,9], isoform-specific differences in SAA function have not been rigorously investigated. Though human SAA1 and SAA2 as well as mouse SAA1.1 and SAA2.1 share high amino acid sequence identity, only human SAA1 and mouse SAA1.1 are deposited into amyloid fibrils [10,11].

The major site of synthesis of SAA is considered to be the liver [12,13,14]. However, extrahepatic expression of SAA has been reported in several different species, including humans, mice, and rabbits [15]. SAA mRNA and protein were reported to be expressed widely in many human tissues, including stomach, small and large intestine, breast, prostate, thyroid, lung, pancreas, kidney, and brain neurons [16]. Adipose tissues express SAA isoforms, and it is reported that, in obesity, adipocytes express more SAA than hepatocytes [17,18]. In mice, SAA3 is an acute-phase reactant expressed by hepatocytes, adipocytes, and, to a lesser extent, macrophages [19,20,21]. In normal healthy states, plasma SAA levels are low. However, chronically elevated SAA is found in a wide variety of pathological conditions, including obesity, rheumatic diseases, cancer, and cardiovascular disease [1,22]. Presence of the SAA genes in human atherosclerotic lesions has been demonstrated by Meek et al. as early as 1994 [23]. Whether SAA plays a direct role in the pathogenesis of these chronic inflammatory diseases, rather than simply being a marker of inflammation, has been a topic of intense investigations over the past several decades.

## 2. SAA Biology

### 2.1. Regulation of SAA Production

Pro-inflammatory cytokines such as Interleukin-1β (IL-1β), Interleukin-6 (IL-6), interferon-γ (IFN-γ), and tumor necrosis factor-α (TNF-α) serve as regulators in hepatic production of SAA during the acute-phase response [1,12]. SAA is also induced by inflammatory stimuli in other cell types, including adipocytes, muscle cells, and intestinal cells [24]. IL-6 seems to be the most critical stimulus of SAA early in the acute-phase response, but the combined activity of all factors gives the highest level of transcription [1]. IL-6 acts via gp130/STAT3 in hepatocytes to induce SAA [25]. However, mice deficient in IL-6 can produce at least a partial acute-phase response, depending on the stimulus [1]. Cytokines other than IL-6 also share a gp130 receptor and may compensate for IL-6 deficiency [1]. A glucocorticoid-responsive element (GRE) is found in human SAA1 but not SAA2 [1]. Consistently in hepatocytes, SAA1, and not SAA2, was found to be preferentially stimulated by dexamethasone; however, cytokine-driven induction is required for this stimulation [26]. Thorn et al. [27] reported that the acute-phase SAA genes are subject to regulatory constraints that differ according to cell type. A variety of transcription factors, including NF-kB, C/EBP, YY1, AP-2, SAF, Sp1, and STAT3, are involved in the induced expression of the acute-phase response resulting in increased SAA production [12].

### 2.2. The Current Challenges and Controversies in the Study of SAA

Understanding the true physiological functions of SAA has been met with several challenges. The majority of the studies investigating the biological activities of SAA have been performed in vitro using recombinantly expressed SAA (rSAA). However, in recent years there have been reports that indicate the unreliability of such proteins, adding to the complexity of understanding SAA. Studies have reported a discrepancy between rSAA of bacterial origin and endogenous SAA, purified from acute-phase plasma. Bjorkman et al. and Christenson et al. observed a lack of inflammatory capacity with endogenous SAA when compared to rSAA of bacterial origin [28,29]. There is also skepticism on the pro-inflammatory activities of the E. coli-derived rSAA, suspecting that LPS contamination may be a major contributing factor to the observed cytokine-like activities. Given the lipid-binding propensity of SAA, LPS may be difficult to separate from the rSAA proteins. Although the LPS content of most of the manufacturer’s products is below 1 ng/mg of SAA protein, an effect of contaminating LPS cannot be excluded, especially considering that TLR4 is one of the SAA receptors. Certain in vitro studies indicate similarities in biological properties shown by endogenous SAA and rSAA. The rSAA (a chimeric protein comprised of hSAA1 with three amino acid replacements/additions from PeproTech Inc., Rocky Hill, NJ, USA) showed potent antiapoptotic effects by decreasing caspase-3/7 activities, and activity was also demonstrated by endogenously purified SAA [29].

The presence of multiple isoforms is the cause of another primary challenge in the field. In part, as SAA3 is a pseudogene in humans, many studies have neglected SAA3 when determining the biological effects of SAA. For example, De Beer et al. have reported that the deficiency of endogenous acute phase SAA1.1 and SAA2.1 did not affect atherosclerotic lesions in apolipoprotein E-deficient (apoE^−/−^) mice [30], which was later found to be due to the pro-atherogenic properties of SAA3 in these mice [9]. The presence of multiple receptors of SAA is yet another challenge, as the downstream signaling by the protein may differ based on the type of receptor activities and expression levels in different cell types. SAA has been shown to exert biological effects via multiple receptors, including formyl peptide receptor-like 1(FPRL-1) [31,32,33,34,35,36,37], FPRL-2 [38,39], TLR2 [40,41], TLR4 [33,42], SR-BI [43,44], CD36 [45], RAGE [46,47,48], and LDL receptor-related protein 1 [49].

## 3. HDL Association

A puzzling aspect of SAA biology shown in numerous studies is that forced overexpression of systemic SAA by itself does not evoke an inflammatory response in mice [50,51], raising questions about how SAA can exert a myriad of activities in vitro yet be seemingly inert in vivo.

SAA is a lipophilic apolipoprotein, and lipid-free SAA is generally not detected in plasma. The majority of liver-derived SAA is typically found associated with high-density lipoprotein (HDL) fraction [52,53,54]. During severe inflammation, SAA can become the major apolipoprotein on HDL [24]. The presence of SAA on HDL affects properties of both SAA and HDL. Many of the properties attributed to SAA are lost when SAA is HDL-bound [50,55]. The inert nature of HDL-bound SAA may be the reason why forced overexpression of systemic SAA by itself does not evoke an inflammatory response in mice [51]. In vitro studies have indicated that HDL-bound SAA can be acted upon by remodeling factors, which could destabilize the HDL particle, probably releasing lipid-poor SAA [56]. One such factor is the cholesteryl ester transfer protein (CETP), which facilitates the exchange of triglycerides on triglyceride-rich lipoprotein with cholesteryl ester on HDL. In vitro studies have shown that CETP-mediated remodeling of HDL facilitates the release of lipid-poor SAA from HDL, as well as the transfer of SAA to apoB-containing lipoproteins [54,56,57]. The masking effect of HDL on SAA’s properties could be to protect the host from tissue damage under homeostatic conditions; it seems likely there are mechanisms to blunt systemic SAA’s inflammatory effects unless it is present in the appropriate context. The removal of SAA from HDL (lipid-free SAA) may give rise to a form that is predisposed to change conformation, potentially in multiple ways. SAA protein with altered conformation could have potent biological properties, or it may be prone to aggregation and tissue deposition and have deleterious effects on organ function [58].

In vitro studies have indicated that the presence of SAA on HDL could affect some of HDL’s properties. HDL has a variety of functions, most important being its anti-inflammatory action and its capacity to promote cholesterol efflux. Acute phase HDL (AP-HDL) containing SAA loses its anti-inflammatory properties and becomes pro-inflammatory [59]. Consistently, AP-HDL carrying SAA is unable to inhibit palmitate-induced expression of pro-inflammatory cytokines, while AP-HDL from mice deficient in SAA1.1 and SAA2.1 exhibits comparable anti-inflammatory action as native HDL [60]. Studies using 3T3-L1 adipocytes indicated that AP-HDL has a reduced capacity to promote cholesterol-efflux compared to AP-HDL from mice deficient in SAA1.1 and SAA2.1 [60]. This is apparently attributable to the binding of cell-surface proteoglycans by the SAA on the HDL, which precludes the ability of HDL to function as a cholesterol acceptor.

Whether SAA affects HDL’s ability to efflux cholesterol during inflammation is a subject of controversy. Although inflammation impairs reverse cholesterol transport [61], de Beer et al. have reported that mice lacking SAA1 and 2 exhibit no impairment in reverse cholesterol transport of radiolabeled cholesterol from macrophages to the feces in vivo [62]. However, upon comparison of five inbred mouse strains whose native HDL proteomes differed quantitatively, SAA1 was inversely correlated with ABCA1-dependent cholesterol efflux [63]. Banka et al. reported a dose effect, in that there was a significant SAA-mediated reduction in cholesterol efflux only when the SAA content of HDL reached about 50% of the total HDL protein [64]. Adding to the confusion is the report that SAA promotes cholesterol efflux. Kisilevsky et al. [65,66] demonstrated that lipid-free SAA2.1 and peptides derived from SAA2.1, but not SAA1.1, promoted efflux. SAA was not only shown to function as a ligand for scavenger receptor class B1 (SR-B1) to promote cholesterol efflux [67], but it also inhibited selective cholesteryl ester uptake from HDL particles [68]. The contrasting conclusions from these studies about the impact of SAA on cholesterol efflux are in part attributable to the differences in approach and methodologies. The use of animal models is perhaps the most reliable method currently available to study the biological function of SAA. SAA levels dramatically increase during acute inflammatory states, for example, from baseline levels of less than 1% to greater than 20% of the HDL protein content by 20 h of endotoxin treatment in mice and returning to baseline levels by 50 h [69]. Currently published animal models of SAA include mice deficient in a single SAA isoform (SAA1.1^−/−^, SAA2.2^−/−^, SAA3^−/−^), mice deficient in several isoforms (SAA1.1, SAA2.1 double knockout; SAA1.1, SAA2.1, SAA3 triple knockout), and mice deficient in all four SAA isoforms [9,49,70,71,72]. In addition, there are models of SAA over-expression [51,73].

## 4. Pathophysiologic Roles of SAA

From an evolutionary perspective, remarkable upregulation of SAA during acute inflammation, along with its high degree of conservation through at least 500 million years of evolution, indicates that SAA plays an important survival role in the systemic response to acute injury and infection. However, SAA expression is inappropriately and persistently elevated in chronic inflammatory diseases, which has been associated with increased risk or poor prognosis for numerous chronic diseases, including CVD and cancer [22,74]. Whether SAA is merely a marker of increased risk or plays a direct role in the pathogenesis has been a topic of investigation by several research groups. Collectively, experiments using murine models of altered SAA expression suggest that, more than being just a biomarker of inflammation, SAA appears to play a causal role in the pathogenesis of CVD, including abdominal aortic aneurysms (AAA) and atherosclerosis.

## 5. SAA and Cardiovascular Diseases

### SAA and Abdominal Aortic Aneurysms

According to estimates, 5–10% of men and 1–2% of women 65–79 years of age in the United States are currently living with AAA, and approximately 15,000 will die each year due to AAA rupture. The etiology of AAA is multi-factorial [75,76], including congenital connective tissue abnormalities, vasculitis of the aortic vasa vasorum, and obesity. However, regardless of the cause, clinical management remains limited and involves monitoring AAA size by ultrasound, with a recommendation for surgical repair if the aortic diameter reaches >5.5 cm, when the risk of fatal rupture is estimated at ≥10% per year [77]. Surgical therapies have shown no benefit in the treatment of small aortic aneurysms (<5 cm), as the risk of rupture is comparable to the risks of surgical intervention. This asymptomatic interval of “watchful waiting” provides an opportunity for medical intervention to reduce AAA expansion and, hence, the risk of rupture. Unfortunately, despite multiple clinical trials, no therapy has proven effective in blunting AAA progression. Although a retrospective analysis demonstrated that ACE inhibitors decreased the risk of aneurysm rupture [78], blockade of the renin–angiotensin system (RAS) has not been found to affect the growth of human AAAs in prospective studies [79], highlighting the need to understand the precise mechanisms underlying AAA risk on an individual basis. Thus, there is an urgent need to not only uncover mechanisms underlying AAA expansion but also to identify biomarkers that may be used as a surrogate marker for progression and provide better information than periodic ultrasound imaging alone.

In a widely used animal model, chronic infusion of AngII to hypercholesterolemic male mice (e.g., apoE^−/−^ or LDLR^−/−^ mice) produces progressive abdominal aortic lumen dilation and pathology that closely resembles the human AAA [80]. Major similarities include a pathogenic role for inflammation, similarities in risk factors (e.g., male gender, obesity), and a likelihood of aneurysm rupture with progressive growth.

AngII infusion induces systemic SAA in mice [71], likely due to AngII’s ability to upregulate TNFα and IL-6 through NF-kB activation [81]. Our group reported the key finding that apoE^−/−^ mice deficient in SAA are protected from AngII-induced AAA. We also reported that SAA co-localizes with breaks in the elastin lamina, prominent matrix metalloproteinase activity, and macrophages in aneurysmal tissue of apoE^−/−^ mice chronically infused with AngII [71]. The investigations are underway to discover the mechanisms leading to the SAA-mediated promotion of AAA formation in mice.

## 6. SAA and Atherosclerosis

Chronic elevation of SAA is found in humans with CVD and CVD risk factors [82,83]. Localized and systemic elevations in SAA have been observed in CVD. Increased circulating SAA is associated with CVD mortality [84]. Among patients admitted with a diagnosis of acute myocardial infarction, elevation of serum amyloid A protein at the time of hospitalization predicts a poor outcome [85]. SAA can be a direct mediator in the development and progression of atherogenesis and atherothrombosis [86]. Analysis of inflammatory markers at the site of ruptured plaques in patients with acute myocardial infarction indicated increased SAA levels, and its levels in the lesions were markedly elevated compared with systemic levels. SAA was detected both within the thrombus itself and white blood cells contained therein. The locally elevated levels of SAA indicate that SAA is produced at the site of coronary occlusion either by cells of the atherosclerotic arterial wall or by the white blood cells trapped in the thrombus [87].

Studies in experimental animals have indicated that SAA plays a causal role in the development of atherosclerosis. SAA binds to heparan sulfate proteoglycans (HSPG), and O’Brien et al. showed that atherosclerotic lesions of both apoE^−/−^ and low-density lipoprotein receptor-deficient (LDLR^−/−^) mice contained demonstrable SAA, whose level correlated highly with lesion area, HSPG, and perlecan content [88]. Proteoglycan-mediated lipoprotein retention is thought to be a critical step in atherosclerosis development [89]. Lentivirus-mediated over-expression of SAA1 in male apoE^−/−^ mice resulted in increased inflammatory cell infiltration and increased atherosclerotic lesion development in the whole aorta and the aortic root in chow-fed mice [90]. Our group has shown that repeated injections of adenoviral vector expressing human SAA1 in apoE^−/−^ mice in the immune-tolerant recombination activating gene-1-deficient background increased atherosclerosis [91]. We also demonstrated that even a single injection of the adenoviral vector encoding SAA1, resulting in only a brief elevation of circulating SAA, was sufficient to increase atherosclerosis [91]. However, we reported no reduction in atherosclerosis in the absence of endogenous SAA1.1 and SAA2.1 in apoE^−/−^ (DKO) mice fed with a standard rodent diet or western diet [30]. In a subsequent study, we were able to show that suppression of SAA3 (via anti-sense oligonucleotide) in DKO mice significantly reduced atherosclerosis compared to apoE^−/−^ mice [9]. These results indicate that all acute-phase SAA isoforms have pro-atherogenic properties, and that suppression of the three isoforms of SAA may be necessary for atheroprotection. In a separate study, deficiency of SAA1 and SAA2 in macrophages decreased the atherosclerotic lesion area in the ascending aorta in LDLR^−/−^ mice only in early lesion development [92].

## 7. Possible Mechanisms for SAA’s Role in AAA and Atherosclerosis

Our studies, as well as studies by other groups, have demonstrated that SAA enhances NLRP3 inflammasome activation and activation of IL-1β in cells [55,93,94,95]. SAA-mediated inflammasome activation in dendritic cells and human synovial fibroblasts has been linked to allergic asthma [96] and gout [97], respectively. NLRP3 inflammasomes are demonstrated to play a role in promoting AAA formation [98,99], and deficiency of the NLRP3 inflammasome prevents AAA formation in AngII-infused apoE^−/−^ mice [98]. Dihlmann et al. reported a significantly increased expression of inflammasome components in human AAA tissue compared to normal human aorta [100], and IL-1β is considered a major factor promoting vessel wall degradation in human AAA [101,102]. We have reported that SAA is required for AngII-induced increases in IL-1β secretion in mice [55].

**Figure 2:**
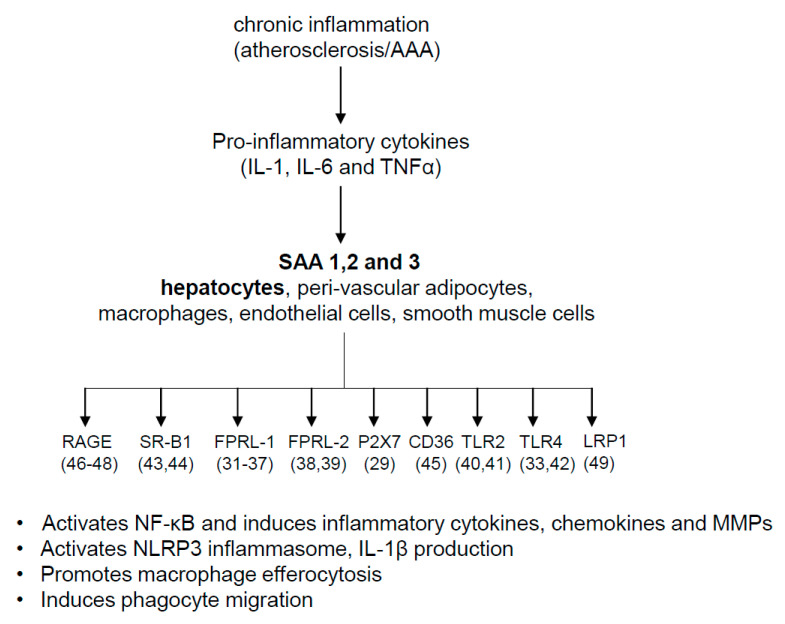
Proposed pathway for the production and activities of acute-phase SAAs in atherosclerosis and AAA. The numbers below the receptor names in parentheses indicate references.

In addition to the activation of NLRP3 inflammasomes, there are several other reported properties of SAA that could potentially enhance the development of AAA formation. Based on in vitro studies, SAA possesses a variety of activities, including cytokine induction [90,103,104], leukocyte chemotaxis [31,105], and upregulation of genes involved in the remodeling of extracellular matrix (ECM), including TGF-β [106] and MMPs [32,107,108,109]. These activities have been attributed to signaling through a number of “pattern recognition receptors” (PRRs) (Figure 2), including formyl peptide receptor-like 1(FPRL-1) [31,32,33,34,35,36,37], FPRL-2 [38,39], TLR2 [40,41], TLR4 [33,42], SR-BI [43,44], CD36 [45], and the LDL receptor-related protein 1 (LRP1) [49]. SAA induces production of tissue factor and tumor necrosis factor in peripheral blood mononuclear cells (PBMC) and immortalized macrophages [103,110] as well as other pro-inflammatory cytokines, such as IL-1β, monocyte chemoattractant protein-1 (MCP-1), IL-6, IL-18, and macrophage inflammatory protein-1 alpha (MIP-1α) in both monocytes and local stromal cells [86,103]. SAA was demonstrated to prolong survival of polymorphonuclear cells by suppressing the apoptotic machinery. The actions were mediated in part through activation of MAPK kinase/ERK and PI3K/Akt signaling pathways, which led to the inhibition of caspase-3 activation, an effect mediated through formyl peptide receptor-like 1/lipoxin A4 receptor activation [111]. Chemotactic potential of recombinant human SAA was first reported by Badolato et al. [112]. Later, several other studies indicated the chemotactic capacity of SAA to multiple cell subsets, including dendritic cells, mast cells, T cells, endothelial cells, fibroblasts, and smooth muscle cells [113]. Several in vitro actions of SAA are known to be mediated through the G protein-coupled FPRL-1 receptor [36,37,114]. Recombinant human SAA stimulates matrix-metalloprotease-9 upregulation via the FPRL1 receptor in human monocytic cells in vitro [32]. SAA may affect key events underlying acute coronary syndromes by contributing to endothelial dysfunction, promoting thrombosis, and enhancing leukocyte trafficking and activation [90,115]. However, many of these above-described properties of SAA were reported from in vitro experiments performed with recombinant or purified SAA and need to be tested in vivo to validate the mechanisms.

SAA is a highly fibrillogenic molecule. Chronically elevated levels of SAA may cause systemic amyloidosis. In vitro studies have shown that SAA can directly bind to fibrin and thus can affect coagulation by promoting amyloid formation in fibrin, and it can also induce platelets to be more prothrombotic [116]. SAA is shown to be a potent inducer of tissue factor from peripheral blood mononuclear cells from patients with coronary artery disease; thus, prothrombotic effects of SAA may contribute to atherogenesis and its complications [86].

## 8. Systemic vs. Local Production of SAA

**Figure 3:**
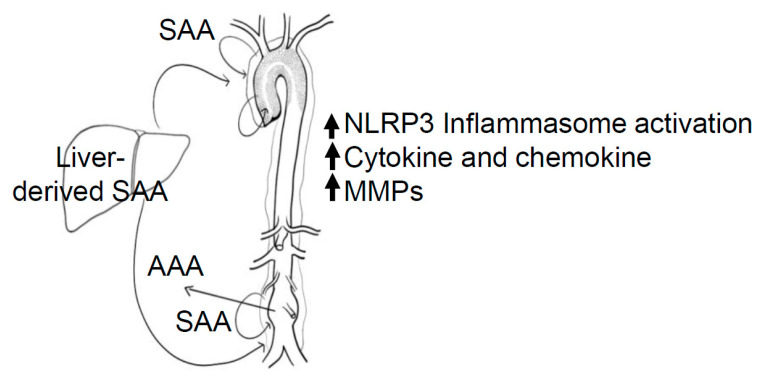
Three possible non-exclusive pathways, whereby SAA impacts the development of atherosclerosis/AAA formation. SAA secreted by the liver, peri-aortic adipose tissues, and/or aorta possibly act in an endocrine, paracrine, or autocrine way respectively by NLRP3 inflammasome activation [55,93,94,95,99] and cytokine, chemokine, and/or MMP expression [32,71,107,108,109], exacerbating the development of atherosclerosis/AAA.

SAA1 and SAA2 are synthesized predominantly in the liver in response to inflammatory stimuli, which contributes to the majority of systemic SAA levels [24]. However, several other cell types, including adipocytes and intestinal cells, also express SAA upon stimulation. The role of local versus systemic SAA is a topic of investigation—SAA may exert effects locally at the sites of tissue injury or inflammation and/or systemically through its presence in circulation (Figure 3). As forced overexpression of systemic SAA does not evoke an inflammatory response in mice, it is logical to assume that HDL—the main transporter of SAA—masks SAA from exerting its inflammatory properties. SAA may only be able to exert effects if unmasked by the remodeling of HDL and deposited at the site of inflammation. In addition, SAA may exert its effects in a paracrine fashion from the cells or tissues that are in close proximity to the site of injury or inflammation. For example, there is a significant amount of perivascular adipose tissue (PVAT) accumulation surrounding the aorta [117]. SAA is persistently elevated in obesity [18,118]. Indeed, adipocytes are thought to be a predominant source of local and even systemic SAA in the setting of obesity [18,118]. Obesity is associated with AAA and increases the risk of cardiovascular-related mortality, and it is a risk factor for the development of AAA in humans as well as in mice [119,120,121,122]. SAA derived from PVAT may contribute to the occurrence or progression of obesity-driven AAA formation. The increased expression of SAA by adipocytes in obesity potentially acts as a direct link between obesity and its comorbidities, including diabetes and cardiovascular diseases [18]. Smoking, a major risk factor for AAA, also induces systemic SAA [123].

## 9. Conclusions

Using animal models, SAA has been shown to play causal roles in the development of CVD, such as atherosclerosis and AAA. As described above, the studies clearly indicate that SAA is not merely a marker for the disease but actively involved in the pathogenesis. Thus, SAA is a potential therapeutic target to consider. However, several important questions still remain in the field including the mechanisms for SAA’s involvement, kinetics of its effect, the source, and systemic or localized expression.

## 10. Key Points

Deficiency/suppression of SAA attenuates atherosclerosis and abdominal aortic aneurysm in mice.

In vitro studies indicate SAA to have proinflammatory properties, and it activates the NLRP3 inflammasome. HDL association of SAA appears to mask its activity.

## Data Availability

Not applicable.
